# A protein scaffold, engineered SPINK2, for generation of inhibitors with high affinity and specificity against target proteases

**DOI:** 10.1038/s41598-019-47615-5

**Published:** 2019-08-07

**Authors:** Daisuke Nishimiya, Yoshirou Kawaguchi, Shiho Kodama, Hatsumi Nasu, Hidenori Yano, Aya Yamaguchi, Masakazu Tamura, Ryuji Hashimoto

**Affiliations:** DAIICHI SANKYO CO., LTD., Biologics Division, Modality Research Laboratories, 1-2-58, Hiromachi, Shinagawa-ku, Tokyo 140-8710 Japan

**Keywords:** Recombinant peptide therapy, X-ray crystallography, Proteases

## Abstract

Proteases are one of attractive therapeutic targets to play key roles in pharmacological action. There are many protease inhibitors in nature, and most of them structurally have cystine knot motifs. Their structures are favorable for recognition of active pockets of proteases, leading to the potent inhibition. However, they also have drawbacks, such as broad cross-reactivity, on the therapeutic application. To create therapeutic proteins derived from a disulfide-rich scaffold, we selected human serine protease inhibitor Kazal type 2 (SPINK2) through a scaffold screening, as a protein scaffold with requirements for therapeutic proteins. We then constructed a diverse library of the engineered SPINK2 by introducing random mutations into its flexible loop region with the designed method. By phage panning against four serine proteases, we isolated potent inhibitors against each target with picomolar K_D_ and sub-nanomolar K_i_ values. Also, they exhibited the desired specificities against target proteases without inhibiting non-target proteases. The crystal structure of kallikrein related peptidase 4 (KLK4)-engineered SPINK2 complex revealed the interface with extensive conformational complementarity. Our study demonstrates that engineered SPINK2 can serve as a scaffold to generate therapeutic molecules against target proteins with groove structures.

## Introduction

Many engineered proteins have emerged as potential therapeutic agents, and have been evaluated in pre-clinical and clinical studies^1–3^. Monoclonal antibodies and antibody-fragment based proteins have wide application as excellent tools for targeting molecules with high specificity. Non-antibody protein scaffolds, including DARPin, Anticalin, and Affibody, were developed, considering that they should overcome drawbacks of antibodies^4–6^. These scaffolds exhibit lower molecular weights than antibodies, and require variable regions to be engineered and stable frameworks, as with antibodies. Using *in vitro* display technologies, such as phage display or ribosome display, engineered proteins with the strong binding affinity are screened^7^. These are artificially engineered proteins, so that the low immunogenicity should be desired for their therapeutic application, as well as the strong binding affinity.

Extracellular proteases can be attractive therapeutic targets, as they are involved in physiologically important cascades; for example, the coagulation cascade and immune responses, among others^8–10^. When proteases deviate from physiological conditions, they can trigger excessive or inappropriate cleavage of substrate proteins, and such dysregulation is implicated in various diseases, including cancer and rheumatism. Proteases are a large family of proteins, approximately one-third of which are serine proteases, which are mainly classified into the S1 family of the mixed nucleophile superfamily A (PA) group in the MEROPS database^11^. Serine proteases have three catalytic residues (His, Asp, and Ser) which are highly conserved^12^. In addition, the active sites of serine proteases have a structurally similar functional groove, known as the catalytic pocket^13^. Given the similarities among their overall structures and catalytic pocket sequences, it is difficult that inhibitors distinguish among these proteases, and to obtain specific inhibitors demonstrating substrate-like binding ability.

Along with the progress of protein engineering, it has been reported that antibodies and Nanobody could be specific protease inhibitors by entirely or partially binding to the pocket of proteases, as well as allosteric inhibition mode^14–18^. On the other hand, natural occurring protease inhibitors can be powerful tools to understand the function of proteases. There are many protease inhibitors such as bovine pancreatic trypsin inhibitor (BPTI) and cystatins in nature^19,20^. Most of them have cystine knot motif forming intra-molecular disulfide bonds, resulting that they show rigid structures and high thermal stabilities^21^. As disulfide-rich proteins have a possibility to form incorrect disulfide bonds which occurs multimeric forms by inter-molecular disulfide bonds, the proper disulfide bond formation is one of the key factors to create therapeutic proteins derived from disulfide-rich scaffolds. In addition, not only the potencies but also the high specificities of inhibitors should be essential. Many natural inhibitors show protease inhibitory activities but also broad cross-reactivities, so that their application as therapeutic tools are limited in terms of side-effects^22^. Indeed, some studies demonstrated that engineering of natural inhibitors such as BPTI and Ecotin improved their specificities^23–25^. Given side-effects resulted from broad cross-reactivities of inhibitors on therapeutic usages, they must selectively recognize just a target protease notwithstanding high similarities among proteases, to reduce adverse effects. Collectively, to create therapeutic engineered proteins, a scaffold should basically have the high efficiency of protein folding and the low immunogenicity. Also, engineered proteins derived from a scaffold require not only potent activities but also as high specificities as possible for therapeutic usages.

Here, we aimed to generate therapeutic proteins targeting proteases with high specificity by engineering a protein scaffold. By screening of scaffolds which met requirements as therapeutic proteins, we selected serine protease inhibitor Kazal-type 2 (SPINK2) as a protein scaffold, followed by constructing an engineered SPINK2 library. Using a series of phage display screening experiments and a diverse engineered SPINK2 library, we succeeded in acquiring quite specific inhibitors for every target protease tested with sub-nanomolar K_i_ values and picomolar K_D_ values. Further, generation of a crystal structure provided insights into the mechanism underlying the high affinity specific binding of those inhibitors for their target molecules, suggesting that the engineered SPINK2 inhibitors preferentially bind to the groove of target proteases via the engineered flexible loop, supported by its constrained structure.

## Results

### Identification of the SPINK2 scaffold as a therapeutic protein

**Figure 1:**
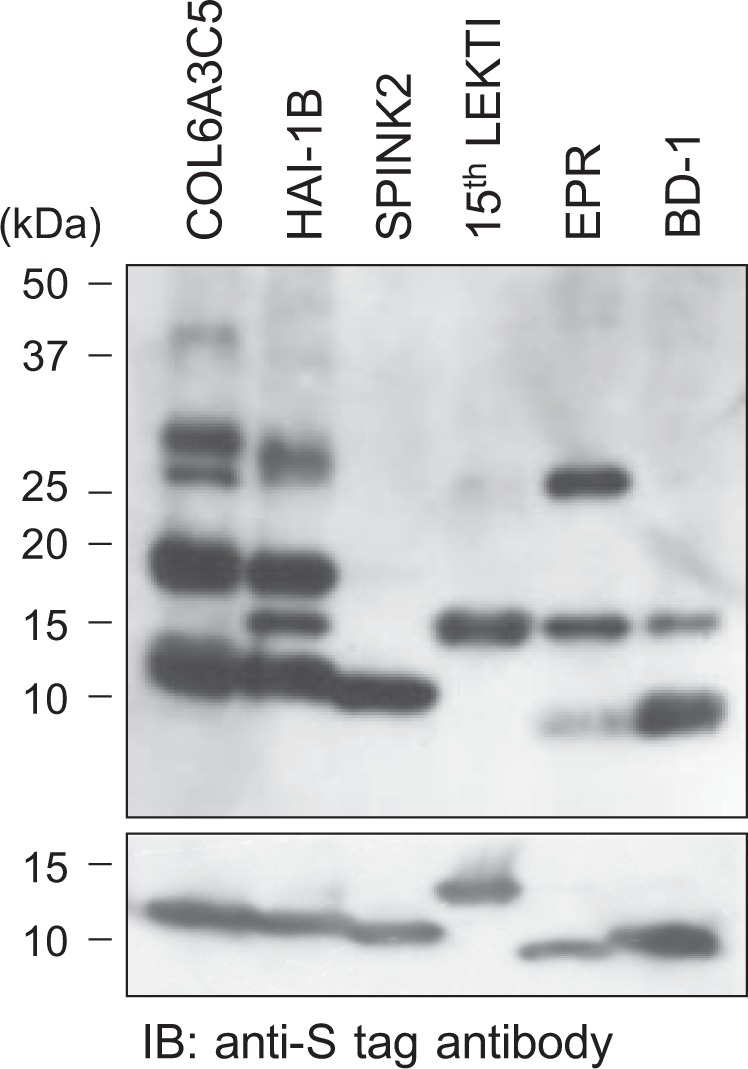
Western blot analysis in scaffold screening. After candidates were expressed in *E.coli* and highly purified, the monomeric forms of candidates were analyzed in SDS-PAGE under (upper) non-reducing and (lower) reducing condition, followed by Western blotting using anti-S tag antibody (Bethyl Laboratories). To ensure equal protein loading in each lane, western blotting analyses under the reducing and non-reducing conditions were performed. COL6A3C5 indicates collagen IV α3 chain C5 domain, HAI-1B indicates HGF activator inhibitor-1 SPINK2 indicates serine protease inhibitor Kazal type 2, 15^th^ LEKTI indicates K Lympho-epithelial Kazal-type-related inhibitor 15th domain, EPR indicates Epiregulin, and BD-1 indicates β-defensin-1.

To obtain a protein scaffold with the properties as therapeutic proteins, we selected six human proteins with cystine knot motif from the Structural Classification of Proteins database (http://scop.mrc-lmb.cam.ac.uk/scop/): collagen IV α3 chain C5 domain (PDB entry 1KTH) and HGF activator inhibitor-1 (PDB entry 1YC0); Kazal-type inhibitors, SPINK2 (PDB entry 2JXD) and Lympho-epithelial Kazal-type-related inhibitor (LEKTI) 15th domain (PDB entry 1UVF); Epiregulin (PDB entry 1K36); β-defensin-1 (PDB entry 1IJV). Regions with cystine knot motif we selected were small (4–9 kDa) and they had three disulfide bonds in common, whereas the disulfide patterns were different. We firstly estimated their folding efficiencies by observing unusual disulfide bond formation in non-reducing SDS-PAGE while they were produced in *E.coli* or displayed on phages. After periplasmic expression in *E.coli* and purification, Kunitz-type inhibitors and Epiregulin showed various bands, suggesting multimeric forms including dimer and trimer by inter-molecular disulfide bonds. β-defensin-1 slightly showed a dimeric form other than a monomeric form, whereas SPINK2 and LEKTI 15th domain did exhibit monomeric forms (Fig. 1). Similarly, SPINK2 and LEKTI 15th domain displayed on phages mainly showed monomeric forms fused to bacteriophage M13 gIII protein, although others contained multimeric forms showing inter-molecular disulfide bonds (Supplementary Fig. 1). These results indicated both Kazal-type inhibitors tend to form a monomeric form with proper intra-molecular disulfide bonds in *E.coli*. Then, we measured the thermal stabilities of purified SPINK2 and LEKTI 15th domain by differential scanning calorimetry (DSC). Both proteins showed high thermal stabilities, but SPINK2 showed higher thermal stability than LEKTI 15th domain: transition temperature (Tm) of SPINK2 and LEKTI 15th domain were 98 °C and 75 °C, respectively (Supplementary Fig. 2). Considering that Tm of antibodies were around 80 °C^26^, it followed that SPINK2 was a more stable scaffold. As the result of scaffold screening, we selected SPINK2 which was suitable for a protein scaffold to be engineered. Furthermore, *in silico* immunogenic prediction score of SPINK2 showed quite low score, EpiMatrix score −34.4, that meant low risk of the immunogenicity, resulting that SPINK2 had the potential for a therapeutic protein.

### Design of the SPINK2 library

**Figure 2:**
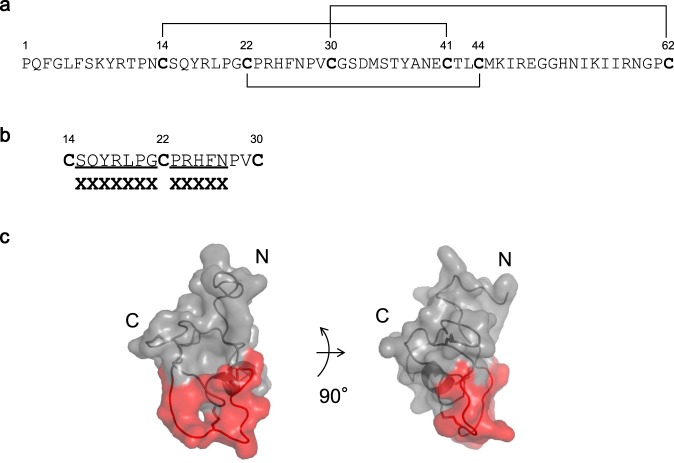
Design of a randomized region to create the engineered SPINK2 library. (**a**) Amino acid sequence of wild type SPINK2. Lines indicate disulfide bonds (Cys14–Cys41, Cys22–Cys44, Cys30–Cys62). (**b**) Region randomized to create the engineered SPINK2 library. (**c**) Three-dimensional structure of wild type SPINK2 (PDB entry, 2JXD). SPINK2 is shown as a *gray* and *red* semi-transparent surface model; *red* indicates the randomized region. The right figure represents the left image turned 90° counterclockwise about the y axis.

SPINK2 comprises a constrained region containing disulfide bonds, and a flexible loop region. The flexible loop is a predicted trypsin binding region^27^, and we generated an engineered SPINK2 library by randomizing this region (Fig. 2). In our preliminary experiments, we investigated residues to be randomized in the flexible loop region, to manage the folding efficiencies of SPINK2. Every Cys residue was fixed without mutations, because the substitution of Cys 22 resulted in the failure of expression (data not shown). In addition, amino acid substitutions at Pro 28 and Val 29 caused incorrect disulfide bonds (data not shown). To maximize the diversity of a library, we therefore selected 12 residues, other than Pro 28 and Val 29 and three Cys residues involved in disulfide bonds, within the loop region to be randomized. To make the ratio of amino acids to be randomized in equal proportions, we applied oligonucleotide-directed random mutagenesis using trimer phosphoramidites. As positions of Cys in SPINK2 were crucial to retain the folding efficiencies, trimer codon mixtures utilized for library construction excluded Cys. Additionally, Pro, of which phi and psi angles are limited, possibly have an influence on the angle of the main chain in a loop region, so that we also excluded Pro from trimer codon mixtures. As a result, the engineered SPINK2 library (theoretical diversity; about 1.0 × 10^15^) constructed by the random mutagenesis using these designed oligonucleotides, yielded 1.2 × 10^10^ independent colonies, indicating substantial functional diversity. The diversity of the engineered SPINK2 library was estimated by deep sequencing using the Illumina HiSeq platform. Among approximately 2.0 × 10^6^ total read sequences at this evaluation, the duplicates ratio was 0.05% and the proportion of clones having inappropriate SPINK2 scaffold sequences 0.57%. This data supported that almost the entire engineered SPINK2 repertoire were of the correct length and contained stochastically generated independent sequences. Although HiSeq analysis did not cover the library diversity, the method of mutagenesis used does achieve generation of extremely diverse libraries.

### Isolation of specific or dual inhibitors with the potency against each target protease

Considering, SPINK2 was originally a trypsin inhibitor^27^, to assess the potential of the engineered SPINK2 library, we chose four serine protease targets of different types: chymotrypsin (chymotrypsin-like serine protease), KLK1 (trypsin- and chymotrypsin-like serine protease), and KLK4 and KLK8 (trypsin-like serine proteases)^28^. To obtain specific binders against chymotrypsin, KLK1 and KLK4 from the engineered SPINK2 library, three sequential rounds of phage panning were performed for each target. In addition, to screen for dual binders of both KLK4 and KLK8, four rounds of phage panning were performed by applying both targets alternately. Enrichment for binders was observed in panning with every target, and duplicate binders of each target protease were rarely observed as a result of sequencing binders. By screening for inhibitory activity using the peptidic substrate against each target, we obtained more than 200 unique inhibitors of which IC_50_ values were less than 100 nM. In terms of the potent inhibitory activity, the top four unique inhibitors against each target were selected for the detailed analysis (Table 1).

**Table 1:** Aligned sequences of inhibitors against each protease: chymotrypsin, KLK1, KLK4, and KLK8.

ID	Target	14								22								30
WT	Trypsin	C	S	Q	Y	R	L	P	G	C	P	R	H	F	N	P	V	C
Library	—	C	X	X	X	X	X	X	X	C	X	X	X	X	X	P	V	C
CT-6	Chymotrypsin	C	R	R	W	L	L	P	W	C	T	Y	K	Y	K	P	V	C
CT-7	Chymotrypsin	C	L	W	R	R	H	K	L	C	P	F	K	F	K	P	V	C
CT-12	Chymotrypsin	C	W	R	S	W	R	W	A	C	P	Y	M	Y	K	P	V	C
CT-14	Chymotrypsin	C	S	T	W	R	M	W	G	C	P	W	L	Y	K	P	V	C
K10061	KLK1	C	A	R	N	N	I	V	D	C	F	Y	Y	Y	K	P	V	C
K10062	KLK1	C	D	I	Y	Q	V	D	R	C	W	W	A	S	Q	P	V	C
K10066	KLK1	C	S	V	A	L	R	D	I	C	W	W	T	S	E	P	V	C
K10071	KLK1	C	D	Q	N	K	Y	R	D	C	H	Y	Y	Y	K	P	V	C
K40001	KLK4	C	R	K	Y	E	Y	G	V	C	Q	R	T	Y	L	P	V	C
K40003	KLK4	C	E	L	Y	V	E	D	V	C	Q	R	I	F	K	P	V	C
K40004	KLK4	C	E	H	A	Q	L	G	V	C	Q	K	L	Y	Q	P	V	C
K40005	KLK4	C	S	Q	Q	A	M	G	A	C	Q	R	I	Y	K	P	V	C
K41043	KLK4/8	C	R	R	Y	S	I	H	G	C	N	R	M	Y	A	P	V	C
K41045	KLK4/8	C	R	K	Q	Y	W	V	G	C	N	R	M	Y	A	P	V	C
K41046	KLK4/8	C	G	R	Y	Y	R	G	W	C	F	K	S	L	E	P	V	C
K41047	KLK4/8	C	M	R	F	H	K	D	G	C	A	R	I	Y	D	P	V	C
												*						

The asterisk (*) indicates the P1 position of K41043.

**Figure 3:**
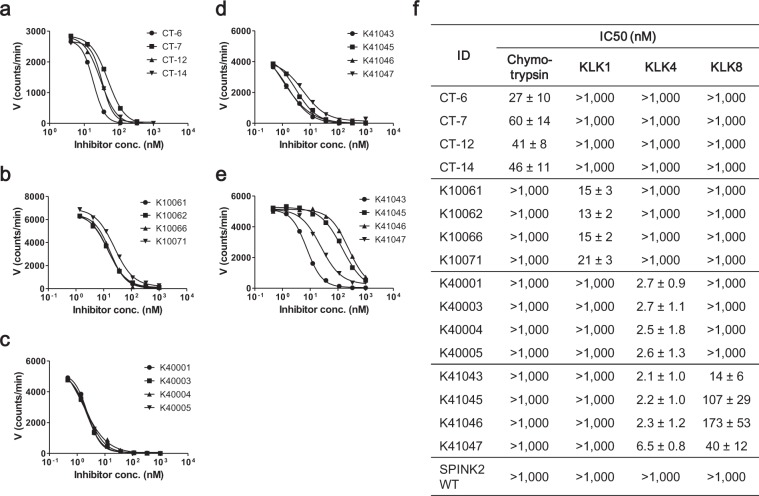
The inhibitory activities of chymotrypsin, KLK1, KLK4, and KLK8 binders. Enzymatic assays of (**a**) chymotrypsin, (**b**) KLK1, and (**c**) KLK4 with selected specific inhibitors, and of (**d**) KLK4 and (**e**) KLK8 with KLK4/8 dual inhibitors. Three independent experiments were performed, and one of three was shown as figures. (**f**) IC_50_ values determined from three independent experiments by sigmoidal nonlinear regression logistic four parameter analysis using GraphPad Prism 5.0 (GraphPad software).

The inhibitors identified by screening were expressed in *E. coli*, then highly purified as monomeric proteins by affinity-purification, followed by size-exclusion chromatography. In enzyme assays of all purified inhibitors, sigmoidal curves were obtained for their target proteases (Fig. 3a–e). The minimum IC_50_ values of inhibitors for their targets were as follows: chymotrypsin inhibitor CT-6, IC_50_ = 27 nM; KLK1 inhibitor K10062, IC_50_ = 13 nM; and KLK4 inhibitor K40004, IC_50_ = 2.7 nM (Fig. 3f). In addition, the KLK4/8 dual inhibitor, K41043 had IC_50_ values of 2.1 and 14 nM for KLK4 and KLK8, respectively (Fig. 3f). These data demonstrate that all inhibitors could inhibit each target with single- or double-digit nM IC_50_ values. The K_i_ values of chymotrypsin, KLK4, and KLK4/8 dual, inhibitors were determined by fitting the Morrison equation. These inhibitors exhibited sub-nanomolar K_i_ values, with a minimum K_i_ value of 0.16 nM (Table 2; Supplementary Fig. 3). Next, we evaluated their specificity for other proteases. Wild-type SPINK2 inhibited trypsin, whereas chymotrypsin and KLK1 inhibitors did not cross-react with any of the 11 proteases, including trypsin, at inhibitor concentrations up to 1 μM. Although some KLK4 inhibitors and KLK4/8 dual inhibitors inhibited trypsin activity, they did not cross-react with the majority of proteases assessed (Supplementary Table 2). These data strongly suggest that the inhibitors obtained in this study exhibit high specificity for their target proteases.

**Table 2:** Activity of each inhibitor against chymotrypsin and KLK4.

ID	Protease	K_i_ (nM)
CT-6	Chymotrypsin	0.17 ± 0.05
CT-7	Chymotrypsin	1.52 ± 0.05
CT-12	Chymotrypsin	0.35 ± 0.07
CT-14	Chymotrypsin	0.59 ± 0.06
K40001	KLK4	0.16 ± 0.03
K40003	KLK4	0.20 ± 0.05
K40004	KLK4	0.38 ± 0.18
K40005	KLK4	0.21 ± 0.07
K41043	KLK4	0.21 ± 0.02
K41045	KLK4	0.32 ± 0.09
K41046	KLK4	0.31 ± 0.05
K41047	KLK4	1.38 ± 0.17

*Results are means ± S.D. for three experiments.

To observe specificities of the inhibitors, three KLK4 inhibitors were selected for binding affinity assessment. To calculate their binding affinity, these inhibitors were analyzed at multiple concentrations and the global kinetic fit evaluated by BIAcore; the results demonstrated fast on-rates and slow off-rates. Consistent with the K_i_ values, their dissociation constants (K_D_) against KLK4 were extremely low (picomolar order); the lowest K_D_ value was 2.1 pM (Table 3; Supplementary Fig. 4). These data suggest that the inhibitors derived from the engineered SPINK2 library exhibited high potency and high specificity against their target proteases.

**Table 3:** Binding affinity of KLK4 inhibitors to KLK4.

ID	k_on_ (M^−1^ s^−1^)	k_off_ (s^−1^)	K_D_ (pM)
K40001	6.99 × 10^6^	1.45 × 10^−5^	2.08
K40003	4.40 × 10^6^	1.02 × 10^−4^	23.2
K41043	1.16 × 10^7^	1.03 × 10^−4^	8.91

### Crystal structure of KLK4 and the KLK4 inhibitor, K41043

**Figure 4:**
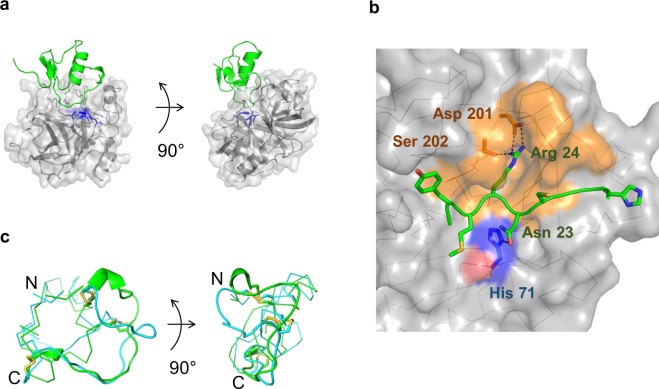
X ray crystal structure of KLK4 and its inhibitor, K41043. (**a**) Structure of the entire KLK4–K41043 complex showing that K41043 interacts with the catalytic pocket residues of KLK4. The light *gray* semi-transparent surface and cartoon model indicates KLK4; the catalytic residues are colored in *blue*. The *green* cartoon indicates K41043. The right figure represents the image on the left turned 90° counterclockwise about the y axis. (**b**) Details of K41043 interaction at the KLK4 catalytic pocket. K41043 residue Asn23 interacts with the catalytic His71 of KLK4, and K41043 residue Arg24 is deeply buried in the S1 pocket. (**c**) Structure of K41043 superposed on to wild type SPINK2. The left figure represents the model on the right turned 90° counterclockwise about the y axis. The letter N indicates N-terminus, while C indicates the C-terminus of K41043.

To investigate the interaction of the K41043 inhibitor with its target, KLK4, at the atomic level, we determined the crystal structure of the KLK4–K41043 complex (PDB entry: 6KBR). A total of 223 residues in the KLK4 model (amino acids 31–253) and 55 residues in the SPINK2 derivative K41043 (amino acids 12–66) were confirmed (Fig. 4a; Supplementary Table 3). The randomized region of K41043 directed to the catalytic pocket of KLK4, and K41043 residue Asn23 formed a hydrogen bond with the catalytic His71 of KLK4 (Fig. 4b). The S1 pocket of KLK4 was deeply buried by K41043 residue Arg24, forming a salt bridge and hydrogen bonds (Supplementary Table 4). This Arg24 was highly conserved among engineered SPINK2-derived inhibitors against trypsin-like serine proteases and/or their substrates (Table 1). The omit map suggested that K41043 was an inhibitor, but not a substrate, under the crystallization conditions (Supplementary Fig. 5). There were few conformational differences in KLK4 with or without K41043; the root-mean-square deviation value between them was 0.346 Å from 190 Cαs. In contrast, there were conformational differences between wild-type SPINK2 and K41043, which were primarily observed the in randomized region; the maximum distance between them was 11.4 Å (Cαs). In addition, K41043 formed a 3_10_-helix at the randomized region of the crystal structure, whereas this was not observed in the wild-type SPINK2 (Fig. 4c). The structural diversity of the randomized region may contribute to its binding to various target molecules. In KLK4, there was 863.3 Å^2^ interacting surface area, composed of residues within a 4 Å distance from the randomized region of K41043. This region contained both S1 pocket and the surface region with a highly variable amino acid sequence (Fig. 4a). The binding loop of K41043 was entirely buried in this large area, providing a high level of conformational complementarity, and there were no water molecules within 7 Å of the catalytic His71 of KLK4 (Fig. 4b). This extensive interaction surface with high complementary conformation could contribute to the potency and specificity of K41043.

## Discussion

In this study, we selected the SPINK2 scaffold through the screening of human proteins with cystine knot motif as a therapeutic tool. We then constructed an engineered SPINK2 library which was functional and diverse, by the combinatorial design to retain the folding efficiency of SPINK2 when introducing randomized amino acids. As a result of phage panning against different proteases, the majority of the inhibitors obtained from the library exhibited sub-nanomolar inhibitory activities, and extremely high specificity without any additional engineering, such as affinity maturation. Owing to the diversity of the library, inhibitors with unique amino acid sequences were obtained against different proteases, despite the structural similarities of the targets. The result of an X ray crystal structure provided deep insight into the mechanisms underlying the high affinity and specificity of the inhibitors. The structure indicates that engineered SPINK2-derived inhibitors bind not only to the S1 pocket, but also around the pocket in a shape complementary manner, and with an extensive target surface. Overall, we successfully validated the potential of engineered SPINK2 as a novel therapeutic protein scaffold for generation of protease inhibitors targeting the groove structure.

**Figure 5:**
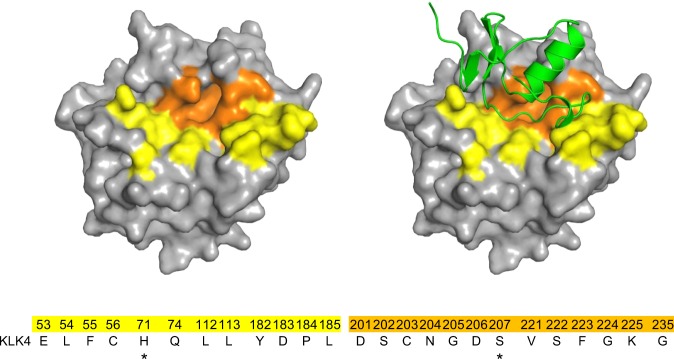
Interaction surface between KLK4 and K41043. The residues within 4 Å of SPINK2 in the molecular surface of KLK4 are colored in *yellow* and *orange*; *orange* colored region are within the S1 pocket. The *green* cartoon model indicates K41043. The colored amino acid numbers and residues are shown below. Asterisks (*) indicate catalytic residues of KLK4.

Proteases, and particularly their active sites, are highly conserved in various organisms^20^. As represented by endopeptidases, proteases tend to recognize the primary sequence in their substrate proteins, where the peptide bond is cleaved^29^. Hence, they may evolve groove structures at their active sites. Conversely, naturally occurring protease inhibitors, such as BPTI, may have also evolved to fit to the active site by developing loop structures^19,30,31^. Cystine knot motif is the most abundant structure in natural protease inhibitors^19^, so that it follows that the shapes of cystine knot proteins are favorable for the protease inhibition. Actually they have a potential for the potent inhibitory activities against proteases, but not every cystine knot protein has properties required for therapeutic proteins. Therapeutic application of proteins requires excellent properties: the protein folding, the stability, the immunogenicity, the inhibitory activity, the specificity, and so on. In accordance with their criteria, we first screened the scaffolds to generate therapeutic proteins derived from cystine knot proteins. Though six candidates used in our study showed similar molecular sizes and structures, there was the apparent difference in disulfide bond formation (Fig. 1, Supplementary Fig. 1). It suggests that the multimeric forms, most of candidates exhibited in expression and phage display studies, should lead to the inefficiency of production or phage panning. We therefore selected SPINK2 which was superior in the folding efficiency. Additionally, the engineered SPINK2 library was constructed with the combinatorial design, resulting in high hit rates to obtain potent inhibitors with high specificities against target proteases. From the structural point of view, the flexible loop of SPINK2 is favorable for interaction with target proteins^27^, and the constrained structure may contribute to biological stability, permitting the loop to move flexibly, with moderate limitation. Indeed, the randomized loop of K41043 entirely covered the active pocket of KLK4, demonstrating that the randomized loop of engineered SPINK2 can provide high shape complementarity at the KLK4–K41043 complex interface (Fig. 5). These data support that engineered SPINK2 can present the loop region, allowing access to protease grooves.

The specificity of protease inhibitors is one of key factors for therapeutic usages. As a target protease, we selected chymotrypsin whose substrate specificity was different from trypsin which was originally a target molecule for the wild type of SPINK2^27^. Similarly, from a structural point of view, we selected KLK1, KLK4, and KLK8 as accessible target proteases. KLK family is one of the largest families among serine proteases, and they show conformational similarity although their amino acid sequences did not exhibit high homology^32,33^. We selected KLK1 with Kallikrein loop as a classical KLK, KLK4 which does not have Kallikrein loop, and KLK8 with a short Kallikrein loop^33^. In isolation of specific inhibitors against chymotrypsin, KLK1, and KLK4, successful screening resulted in inhibitors, all of which exhibited high affinity and specificity for their target proteases. These results suggest that engineered SPINK2 has the potential to bind and inhibit any type of serine protease. In addition, we challenged the screen to generate dual inhibitors against KLK4 and KLK8. The generation of dual, in addition to specific, inhibitors would have high impact on research and/or therapeutic fields, since it can produce single drugs that function against multiple targets. Both KLK4 and KLK8 are classified as trypsin-like serine proteases, and the sequence similarity between these two proteins is 79%. As KLK4 specific inhibitors were successfully obtained, we tried to obtain dual inhibitors against KLK4 and KLK8, which was in activation cascades^34^. We also succeeded in isolating dual inhibitors which showed strong inhibition of both KLK4 and KLK8 with a panning strategy using each protease alternately.

The engineered SPINK2-derived inhibitors contained amino acids to mimic P1 residues of the protease recognition site, in a similar manner to substrates (Table 1). In the crystal structure, Arg24 of K41043, corresponding to the P1 position of the substrate, was deeply buried in the S1 pocket of KLK4 (Supplementary Fig. 6). As trypsin-like serine proteases have deep S1 pocket with an acidic residue at the base, residues such as Lys or Arg are preferable at the P1 position of substrates^35–37^. Chymotrypsin has a deep hydrophobic pocket, hence residues such as Phe, Tyr, or Trp are desirable at the P1 position^38^. Consistent with such selectivity, KLK1, KLK4, and KLK4/8 dual inhibitors have Arg or Lys, while chymotrypsin inhibitors contain residues with aromatic rings, at the position corresponding to substrate P1 site. These data support that all engineered SPINK2-derived inhibitors may bind in a substrate-like manner. In the KLK4–K41043 co-crystal structure, there were no water molecules within 7 Å from the catalytic His71 of KLK4. This may be because binding of K41043 to KLK4 excluded the water molecules essential for protease turnover, hence K41043 acted as an inhibitor, but not as a substrate. Indeed, the Fo-Fc omit map of K41043 was not interrupted in the crystal structure, which strongly supports that K41043 is not a substrate, but rather an inhibitor (Supplementary Fig. 5). These data also indicate that the engineered SPINK2 library should provide not only “binders”, but also “inhibitors”.

The crystal structure of the KLK4–K41043 complex further illuminates the mechanism underlying the high affinity and specificity of the binding of engineered SPINK2 to KLK4. It revealed that 25 residues of KLK4 within 4 Å from K41043 were located not only in the S1 pocket, including the catalytic residues (His71 and Ser207), but also in a surface distant from the S1 pocket (Fig. 5). Although the primary sequences of the contact residues in the S1 pocket are conserved among serine proteases, the sequence and number of amino acids in the interaction surface distant from S1 pocket exhibit exceedingly high diversity (Fig. 4a, Supplementary Table 5). Their interaction surface is located in the functional groove, the so-called active pocket; therefore, it is logical that these grooves exhibit structural similarity. Nevertheless, the sequences of proteases vary, despite the conserved S1 pocket, because they must distinguish among substrates to fulfil their distinct physiological roles^13^. In the KLK4–K41043 structure, the entire interaction surface area was 863.3 Å^2^, and included salt bridges, hydrogen bonds, and hydrophobic contacts (Supplementary Table 4). In addition to these multiple interactions, the loop complementarity with the pocket of the target may have contributed to van der Waals interactions.

The fact that inhibitors with various sequences in their loop regions exhibited different specificities suggests that contact regions with target proteases involved their randomized loops. Furthermore, considering the preference of the P1 residues of inhibitors, which correspond to their target proteases, inhibitors other than K41043 are also likely to exhibit similar interaction characteristics, with recognition of the extensive surface, composed of the conserved S1 pocket and the more diverse surface region. Regarding binding to targets, the importance of the interaction area outside of the S1 pocket is also indicated by studies using bovine pancreatic trypsin inhibitor (BPTI). BPTI exhibits exceedingly strong trypsin inhibition with a K_i_ of 0.06 nM, and the Lys residue at its P1 position is buried in the S1 pocket^39^. Meanwhile, BPTI can also inhibit chymotrypsin with a K_i_ of 9 nM^39^. In the chymotrypsin-BPTI complex crystal structure (PDB entry, 1CBW), the surface area outside of the S1 pocket is accessed by BPTI, although the P1 residue is not buried in the S1 pocket; the Lys of the P1 residue is not oriented to the Ser at the bottom of the S1 pocket in chymotrypsin^40^. The P1–S1 interaction is clearly important to recognition of a target protease; however, the additional region around S1 pocket is also required to inhibit the target activity. Our study demonstrates the significance of the extensive interaction surface, including the protease S1 pocket, for potent and specific inhibition. In addition, we were able to obtain both extremely high specificity inhibitors and dual inhibitors, such as K41043, from the diverse library, indicating that individual amino acids in inhibitors may also make strong contributions to the selective binding of target molecules, through optimization of the shape complementary interaction; for example, by conferring minor conformational adjustments. Hence, engineered SPINK2-derived inhibitors recognize the functional groove of proteases through extensive binding around the S1 pocket in a conformation complementary manner, leading to their high specificity and potent inhibitory activity.

In conclusion, we identified SPINK2 scaffold with the properties necessary for therapeutic proteins through screening of human cystine knot proteins, and then generated a novel protein scaffold derived from SPINK2 to achieve highly potent and specific inhibition of target proteins with surface grooves. The engineered SPINK2-derived inhibitors bind in a unique manner, where the randomized loop creates a large interface with a high degree of conformational complementarity between the loop and the groove of the target protein. This manner of binding suggests that target molecules should not be limited to proteases, but extend to other proteins containing structurally similar grooves; therefore, the engineered SPINK2 scaffold is a potentially powerful therapeutic tool.

## Methods

### Differential scanning calorimetry (DSC)

DSC was conducted a MicroCal VP-Capillary DSC (Malvern Instruments) at a heating rate of 60 °C/h. All samples were tested at a concentration of 0.25 mg/mL in phosphate buffered saline (PBS), and they were heated from 20–110 °C. Transition midpoint values (Tm) were determined using the software MicroCal Origin 7 (Malvern).

### Construction of a SPINK2 library

The oligonucleotide encompassed the region encoding residues Ser 15 to Asn 27 of SPINK2 (Uniprot; P20155) which was to be randomized (5′-GC AAA TAT CGT ACC CCG AAT TGT XXX XXX XXX XXX XXX XXX XXX TGT XXX XXX XXX XXX XXX CCG GTT TGT GGT AGC GAT ATG-3′) was synthesized using trimer phosphoramidites (TSUKUBA OLIGO SERVICE). A non-randomized region of *SPINK2* was amplified using the primers forward, 5′-GGTAGCGATATGAGCACCTATGC-3′, and reverse, 5′-GCACGGACCATTGCGAATA-3′. Next, an overlap PCR was performed using the synthesized oligonucleotides, amplified fragment, and the primers forward, 5′-AAAGAATTCTGATCCGCAGTTTGGTCTGTTTAGCAAATATCGT-3′; reverse, 5′-AAAGGCGCGCCGCACGGACCATTGCGAATAATTTTAAT-3′. The resulting PCR product was sub-cloned into the modified phagemid vector, pCANTAB 5E (GE Healthcare), which contained (in order): a region encoding the phoA signal peptide; the SPINK2 library; a TEV recognition site for cleavage with TEV protease; and gene III, encoding protein gIII of bacteriophage M13. After digestion of DNA with restriction enzymes, the ligated DNA was transformed into *Escherichia coli* XL1-Blue by electroporation, yielding 1.2 × 10^10^ independent colonies.

### Next-generation sequencing (NGS)

The phagemid DNA from the SPINK2 library was analyzed using a HiSeq system (Illumina Inc.). The HiSeq library for DNA sequencing was prepared using a Nextra XT index kit (Illumina Inc.) following the protocol provided by the manufacturer. To amplify DNA for NGS, the PCR reaction was performed using the primers forward, 5′-TCGTCGGCAGCGTCAGATGTGTATAAGAGACAGGATCCGCAGTTTGGTCTGTTTAGC-3′ and reverse, 5′-GTCTCGTGGGCTCGGAGATGTGTATAAGAGACAGGGCCACCTTCACGAATTTTCATG-3′, and Pfu Turbo DNA polymerase (Agilent Technologies). Sequencing data were obtained from TAKARA BIO INC.

### Target proteins and substrates

All substrates and proteins, except for KLK1, KLK4, and KLK8, were purchased from commercial suppliers (Supplementary Table 1). For phage panning, chymotrypsin (Worthington Biochemical Corporation) was immobilized on resin using Pierce NHS-activated agarose (Thermo Fisher Scientific). Expression vectors encoding KLK1 (UniProt; P06870), KLK4 (UniProt; Q9Y5K2), and KLK8 (UniProt; O60259) were constructed with His tags at their C-termini, by introducing sequences into the pcDNA3.3 vector (Thermo Fisher Scientific). Pro-KLK1, KLK4, and KLK8 were produced in FreeStyle 293-F cells (Thermo Fisher Scientific) and purified using His Trap excel columns (GE Healthcare). Each enzyme was activated using thermolysin (Sigma-Aldrich) in activation buffer (50 mM Tris, 10 mM CaCl_2_, 150 mM NaCl, pH 7.5), then their reactions were stopped by adding 10 mM 1,10-phenanthroline (Sigma-Aldrich). After activation, buffers were exchanged for PBS using Amicon-Ultra 15 (10,000 NMWL; EMD Millipore). Biotinylation of proteins was performed using a 4-fold molar excess of EZ-Link NHS-PEG4-Biotin (Thermo Fisher Scientific) in PBS, then biotinylated proteins were purified using Amicon-Ultra 15.

### Phage panning

Phage panning was performed to enrich binders to each target protein; immobilized chymotrypsin, and biotinylated KLK1, KLK4, or KLK8. The SPINK2 library was superinfected with the helper phage VCS M13 (Agilent Technologies), following the standard protocol for construction of a phage-display library^41^. The SPINK2 phage library was cycled through 3–4 rounds of binding selection with each target protein. In the first round, approximately 1.8 × 10^13^ phages displaying mutated SPINK2 were incubated with each target protein in 3% BSA in PBS containing 0.05% Tween 20 at 4 °C. Phages bound to biotinylated targets were collected using Dynabeads® M-280 Streptavidin (Thermo Fisher Scientific). Bound phages were washed several times with PBS containing 0.05% Tween 20. Then, they were eluted using AcTEV™ Protease (Thermo Fisher Scientific) which cleaved the phages between mutated SPINK2 and gIII proteins. The recovered phage repertoire was amplified in XL1-Blue cells, which were then subjected to the following round of panning. During subsequent selection rounds, the number of washing steps was gradually increased, and the antigen concentration was decreased. For selection of KLK4/8 dual inhibitors, the SPINK2 phage library was subjected to biotinylated KLK4 in round 1, followed by biotinylated KLK4 and KLK8 alternately.

### Expression and purification

Gene-of-interests including scaffold candidates and enriched phagemid DNA were cloned into the pET32a vector. Scaffold candidates were as follows; Kunitz-type inhibitors, collagen IV α3 chain C5 domain (UniProt; P12111, 2501–2558) and HGF activator inhibitor-1 (UniProt; O43278, 245–303); Kazal-type inhibitors, SPINK2 (UniProt; P20155, 23–84) and LEKTI 15th domain (UniProt; Q9NQ38, 989–1064); Epiregulin (UniProt; O14944, 63–108); β-defensin-1 (UniProt; P60022, 33–68). Origami B (DE3) were transformed with each expression vector and cultured at 37 °C, then IPTG was added. After cultivation at 16 °C overnight, cell pellets were collected and lysed using BugBuster® Master Mix (EMD Millipore). Protein-of-interests were purified using TALON® Metal Affinity Resins (Clontech), then thioredoxin tags were removed using a Thrombin Cleavage Capture Kit (EMD Millipore). They were finally purified using Superdex-75 (GE Healthcare) size exclusion chromatography, and applied to enzyme assays for binder selection, as described below. Multimeric formation analysis of candidates was confirmed by SDS-PAGE, followed by western blotting analysis: goat anti-S-Tag Antibody HRP Conjugated (Betyl) was used for purified proteins; HRP/Anti-M13 Monoclonal Conjugate (GE healthcare) was used for detection of proteins fused to the bacteriophage M13 gIII protein.

### Enzymatic assays

All enzymatic assays were performed at 37 °C in 96-well black plates (Sumitomo Bakelite) in assay buffer (50 mM Tris-HCl, 150 mM NaCl, pH 8.0), and monitored using an EnSpire™ fluorescence plate reader (PerkinElmer) with excitation and emission wavelengths at 380 and 460 nm, respectively. For protease inhibition assays, binders at various concentrations (0–1,000 nM) was pre-incubated with each protease for 15 min at 37 °C, followed by addition of each fluorescent substrate. Initial reaction velocities were determined by a linear fit to plot, with fluorescence on the ordinate and time on the abscissa axes^42^. Velocities were calculated as the slopes of the regression lines from the time interval 1–5 min, and their values were used to calculate IC_50_ values. IC_50_ values were determined from three independent experiments by sigmoidal nonlinear regression logistic four parameter analysis using GraphPad Prism 5.0 (GraphPad software). K_i_ values were also determined from three independent experiments by fitting the Morrison equation for tight binding inhibitors to the relative reaction velocity using nonlinear regression in GraphPad Prism 5.0^43^. Results are means ± S.D. for three experiments.

### Surface plasmon resonance (SPR) analysis

SPR was measured using a BIAcore T200 (GE healthcare) with HBS-EP running buffer (10 mM HEPES, 150 mM NaCl, 3 mM EDTA, 0.05% (v/v) Surfactant P20, pH 7.4). Streptavidin conjugated DNA was captured on a Sensor Chip CAP (GE healthcare), followed by immobilization of biotinylated KLK4 on its chip at approximately 5 response units (RU). KLK4 inhibitors were subsequently captured by injection of varying concentrations (0.08–20 nM) of KLK4 inhibitors diluted with HBS-EP running buffer for 5 min at a flow of 10 μL/min, and then dissociation measured for 60–360 min with buffer flow. The signal of reference cells was subtracted from the measurements. The kinetic data of the interaction were evaluated with a global fit using BIAcore T200 evaluation software. Reference surface and chip regeneration were performed with regeneration buffer from the Biotin capture kit (GE healthcare).

### Crystallization of KLK4 and SPINK2-derivative complex

Purified KLK4 and its inhibitor (ID; K41043) were mixed and incubated for 1 h at 25 °C. The KLK4–K41043 complex was isolated by gel-filtration chromatography, and concentrated to 30 mg/mL using Amicon-Ultra 15 filter units. To remove the S-tag fused to the N-terminus of SPINK2, EKMax™ Enterokinase (Thermo Fisher Scientific) was added to the complex to a final concentration of 16.7 mU.

Crystallization experiments were performed using the sitting-drop vapor diffusion method at 20 °C. Screening was carried out using PEG/Ion HT crystallization screening kits (Hampton Research) by mixing 500 nL of protein solution with 500 nL of reservoir solution. A crystal was obtained in one day from condition No. 4 of the PEG/Ion HT kit (0.2 M lithium chloride, 20% (w/v) polyethylene glycol 3350). The crystal was soaked in cryoprotectant buffer (20% (v/v) glycerol, PBS, 0.2 M lithium chloride, 20% (w/v) polyethylene glycol 3350) and then flash cooled in liquid nitrogen.

### Determination and analysis of the KLK4–K41043 complex structure

A data set was collected on beamline NE3A at Photon Factory, Tsukuba, Japan. A total of 720 frames of data were collected using a 0.25° oscillation range with 1.5 sec exposure. Data were indexed, integrated, and scaled using iMOSFLM^44^. Molecular-replacement calculations were carried out with Phaser MR, using the three-dimensional structures of KLK4 and wild type SPINK2 (PDB entries 4K1E and 2JXD, respectively), as search models^45,46^. The structure was then revised several times by alternately adjusting the model, and refinements made using COOT and Refmac^47,48^. The figure was generated using the PYMOL program. Interactions between KLK4 and K41043 were analyzed using the Protein Interaction Calculator server^49^.

### *In silico* immunogenicity prediction

Immunogenicity of the scaffold candidates were evaluated using ISPRI program^50^ (EpiVax, Inc.). The sequence of each scaffold was parsed into overlapping 9-mer frames, and the immunogenic potential of each frame was assessed against a panel of eight archetypal HLA class II alleles that represent 90% of MHC diversity in the human population.

## Supplementary information

Supplementary information
