# Supplementary material for: A protein scaffold, engineered SPINK2, for generation of inhibitors with high affinity and specificity against target proteases
Source: Sci Rep. 2019 Aug 7;9:11436. doi: 10.1038/s41598-019-47615-5 (PMC6686015; doi:10.1038/s41598-019-47615-5)
Supplement: Supplementary file 1 — Supplementary information [file 41598_2019_47615_MOESM1_ESM.pdf]

Supplementary information

A protein scaffold, engineered SPINK2, for generation of inhibitors with high affinity and specificity against target proteases

Authors and affiliation:
Daisuke Nishimiya*, Yoshirou Kawaguchi, Shiho Kodama, Hatsumi Nasu, Hidenori Yano, Aya Yamaguchi, Masakazu Tamura, Ryuji Hashimoto

DAIICHI SANKYO CO., LTD., Biologics Division, Modality Research Laboratories
1-2-58, Hiromachi, Shinagawa-ku, Tokyo 140-8710, Japan

Supplementary Table 1. Enzyme assay conditions

**Protease**	**Final conc. of protease**	**Substrate**	**Final conc. of substrate**
Chymotrypsin (Worthington)	10 nM	Suc-LLVY-MCA (3120-v; PEPTIDE INSTITUTE)	10 μM
KLK1	1 nM	H-PFR-AMC (I-1295; Bachem)	100 μM
KLK4	2.5 nM	Boc-VPR-AMC (ES011; R&D systems)	100 μM
KLK8	5 nM	Boc-VPR-AMC	100 μM
TPCK bovine trypsin (PIERCE)	5 nM	Boc-VPR-AMC	100 μM
plasma kallikrein (R&D systems)	0.125 μg/mL	PFR-AMC (3096-v; PEPTIDE INSTITUTE)	100 μM
Tryptase (Sigma)	1 nM	Boc-FSR-MCA (3107-v; PEPTIDE INSTITUTE)	100 μM
Chymase (Sigma)	100 nM	Suc-AAPF-MCA (3114-v; PEPTIDE INSTITUTE)	10 μM
Matriptase (R&D systems)	1 nM	Boc-QAR-AMC (ES014; R&D systems)	100 μM
Thrombin (Sigma)	1 nM	Boc-VPR-AMC	100 μM
Plasmin (Sigma)	50 nM	Boc-VLK-MCA (3104-v: PEPTIDE INSTITUTE)	100 μM
Elastase (ENZO)	10^{-5} unit/μL	Suc(OMe)-AAPV-MCA (3153-v; PEPTIDE INSTITUTE)	100 μM
Protein C (Sigma)	100 nM	Boc-LSTR-AMC (3112-v; PEPTIDE INSTITUTE)	100 μM
tPA (Sigma)	10 nM	Pyr-GR-MCA (3145-v; PEPTIDE INSTITUTE)	100 μM
uPA (Sigma)	2 nM	Pyr-GR-MCA	100 μM

Supplementary Table 2. Cross-reactivity of inhibitors against various serine proteases

The remaining protease activity in the presence of 1 μM of inhibitors is shown, compared with that of buffer control.

ID	Trypsin	Chymo trypsin	Tryptase	Chymase	Plasmin	Thrombin	Elastase	Matriptase	Protein C	tPA	uPA	Plasma kallikrein
**Buffer**	100	100	100	100	100	100	100	100	100	100	100	100
**WT**	0	106	100	109	43	115	194	76	145	185	156	32
**CT-6**	93	0	94	111	133	113	190	112	204	227	176	120
**CT-7**	95	0	79	78	88	100	175	92	108	287	101	84
**CT-12**	87	0	64	60	74	105	129	87	87	226	103	73
**CT-14**	91	0	104	113	138	119	180	122	185	205	161	122
**K10061**	88	52	99	99	113	101	112	102	134	109	127	106
**K10062**	45	77	102	109	115	104	125	104	139	90	130	106
**K10066**	101	99	103	105	115	109	122	104	140	114	130	109
**K10071**	104	23	104	109	116	108	121	105	150	103	130	113
**K40001**	5	96	104	99	92	107	100	105	148	147	161	118
**K40003**	30	79	107	102	111	113	106	108	184	246	163	122
**K40004**	7	102	105	103	103	119	105	109	184	252	167	116
**K40005**	10	102	114	106	114	119	92	109	160	242	163	121
**K41043**	0	95	101	108	12	107	112	47	109	136	113	85
**K41045**	0	104	104	107	6	111	111	105	152	143	119	93
**K41046**	0	108	102	110	3	111	107	107	138	142	117	109
**K41047**	0	103	104	109	61	111	123	109	138	132	117	103

Supplementary Table 3. Crystallographic data collection and refinement of the KLK4–K41043 structure

**Data collection**	**NE3A 2017/04/26**
Space group	*C2*
Cell dimensions	
a, *b*, *c* (Å)	145.21, 41.62, 43.59
a, b, g (°)	90, 91.58, 90
Resolution (Å)	72.6-1.9 (2.0-1.9)
R_{merge}	0.099 (0.245)
I / s*I*	8.8 (4.4)
Completeness (%)	99.6 (98.3)
**Refinement statistics**	
Resolution (Å)	2.0
R_{work} / R_{free}	0.183 / 0.226
R.m.s. deviations	
Bond lengths (Å)	0.0193
Bond angles (°)	1.9775
Number of residues	
favoured	267 (97.45%)
Allowed	7 (2.55%)
Outlier	0 (0%)

Supplementary Table 4. Interactions between KLK4 and K41043 searched using the Protein Interaction Calculator

Hydrogen bonds, salt bridge, and hydrophobic contacts were detected by the Protein Interaction Calculator.

Hydrogen bond

K41043		KLK4	
a.a. Number	residue	a.a. Number	residue
20	H	225	K
22	C	224	G
23	N	71	H
23	N	204	N
24	R	201	D
24	R	202	S
24	R	205	G
24	R	207	S
24	R	222	S
25	M	207	S
26	Y	55	F
39	N	204	N
45	M	226	A
49	E	226	A

Salt bridge

K41043		KLK4	
a.a. Number	residue	a.a. Number	residue
24	R	201	D

Hydrophobic contact

K41043		KLK4	
a.a. Number	residue	a.a. Number	residue
25	M	50	M
25	M	55	F
26	Y	55	F
26	Y	155	L
38	A	155	L
26	Y	161	M
45	M	226	A

Supplementary Table 5. Comparison of KLK4 with other serine proteases

Residues of KLK4 within 4 Å from K41043 are shown at the top of this table. The corresponding sequences of other proteases used in this study are aligned. Completely conserved residues are colored in *black*; over 80% of residues are colored in *gray*. Asterisks (*) indicate the catalytic residues of KLK4.

amino acid number of KLK4	53	54	55	56	71	74	112	113	182	183	184										185	201	202	203	204	205	206	207	221	222	223	224	225	235
KLK4	E	L	F	C	H	Q	L	L	Y	D	P	-	-	-	-	-	-	-	-	-	L	D	S	C	N	G	D	S	V	S	F	G	K	G
KLK8	Q	L	L	C	H	K	D	V	Y	P	G	-	-	-	-	-	-	-	-	-	Q	D	T	C	Q	G	D	S	T	S	W	G	S	G
KLK1	T	F	Q	C	H	S	L	L	H	V	Q	-	-	-	-	-	-	-	-	-	K	D	T	C	V	G	D	S	T	S	W	G	Y	S
bovine Trypsin	Y	H	F	C	H	K	T	L	Y	P	G	-	-	-	-	-	-	-	-	-	Q	D	S	C	Q	G	D	S	V	S	W	G	S	G
Matriptase	R	H	L	C	H	Q	E	D	Y	R	-	-	-	-	-	-	-	-	-	Y	Q	D	A	C	Q	G	D	S	V	S	W	G	L	G
Plasma kallikrein	R	H	L	C	H	-	V	S	Y	Q	D	-	-	-	-	-	-	-	-	Y	K	D	A	C	K	G	D	S	T	S	W	G	E	G
Thrombin	E	L	L	C	H	L	R	E	T	R	-	-	-	-	-	-	-	-	-	I	R	D	A	C	E	G	D	S	V	S	W	G	E	G
Protein C	K	L	A	C	H	D	K	S	M	S	-	-	-	-	-	-	-	-	-	N	M	D	A	C	E	G	D	S	V	S	W	G	E	G
Tryptase	M	H	F	C	H	G	T	A	Y	H	L	G	A	Y	T	G	D	D	V	R	I	D	S	C	Q	G	D	S	V	S	W	G	E	G
uPA	T	Y	V	C	H	I	T	L	H	Y	Y	G	-	-	-	-	-	-	-	S	E	D	S	C	Q	G	D	S	V	S	W	G	R	G
tPA	R	F	L	C	H	Q	T	-	H	L	L	N	-	-	-	-	-	-	-	R	T	D	A	C	Q	G	D	S	I	S	W	G	L	G
Plasmin	M	H	F	C	H	E	-	-	E	F	L	N	-	-	-	-	-	-	-	G	R	D	S	C	Q	G	D	S	T	S	W	G	L	G
bovine Chymotrypsin	F	H	F	C	H	-	S	L	W	G	-	-	-	-	-	-	-	-	-	T	K	S	S	C	M	G	D	S	V	S	W	G	S	G
Chymase	S	K	F	C	H	G	T	S	R	D	F	D	-	-	-	-	-	-	-	-	-	S	A	F	K	G	D	S	V	S	Y	G	R	A
Neutrophil elastase	G	H	F	C	H	A	L	L	-	-	-	-	-	-	-	-	-	-	-	-	L	G	V	C	F	G	D	S	A	S	F	V	R	D
					*																							*						

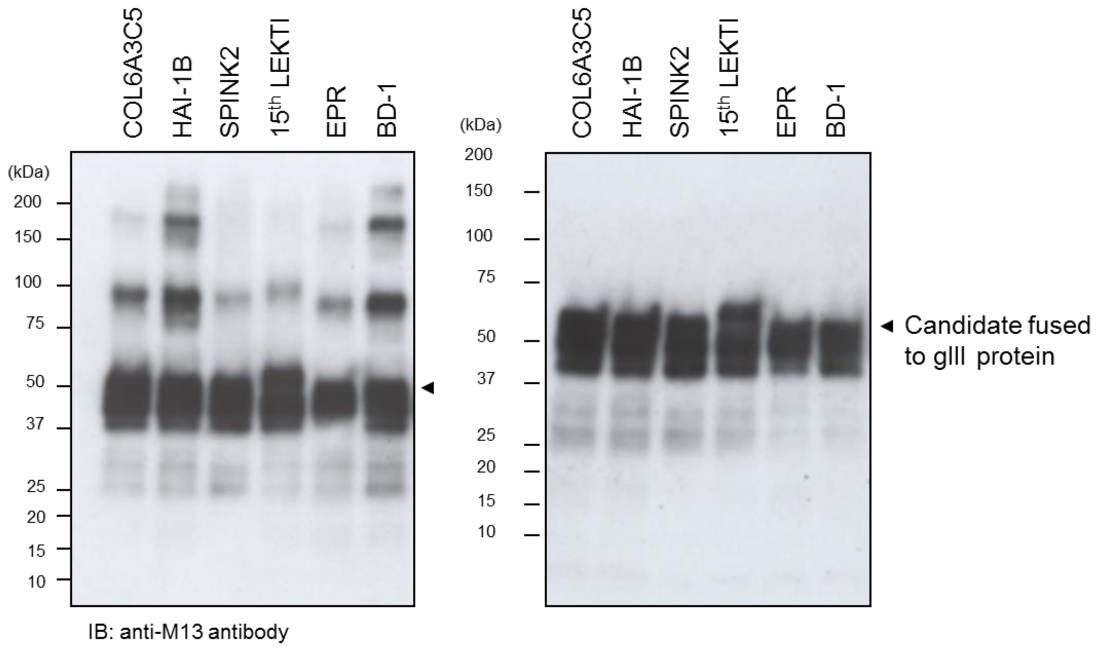

Supplementary Figure 1: Western blotting analysis of candidates fused to the bacteriophage M13 in scaffold screening. After candidates fused to the gIII proteins of the bacteriophage M13 were expressed in *E.coli* and highly purified, the folding efficiencies of candidates were analyzed in SDS-PAGE under (left) non-reducing and (right) reducing condition, followed by Western blotting using HRP/Anti-M13 Monoclonal Conjugate (GE healthcare). To ensure equal protein loading in each lane, Western blotting analyses under the reducing and non-reducing conditions were performed.

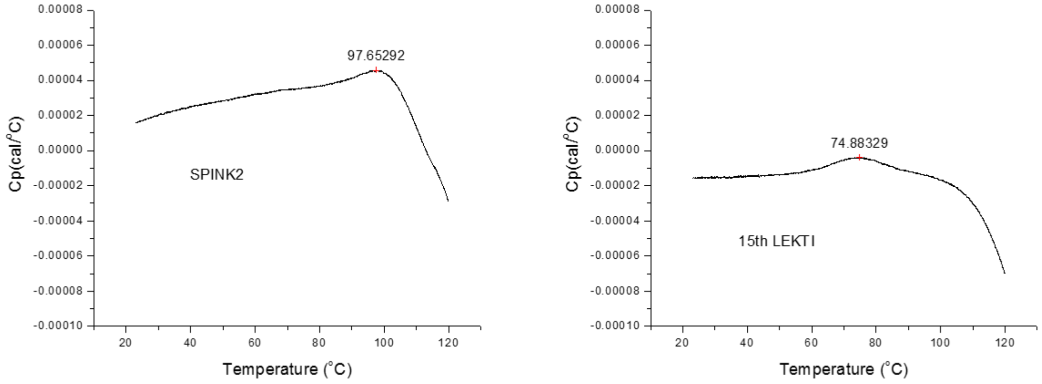

Supplementary Figure 2: Thermal stability of Kazal-type inhibitors by differential scanning calorimetry (DSC). Transition midpoint values (Tm) were determined using the software MicroCal Origin 7. The Tm value of SPINK2 showed approximately 98 °C, and the Tm value of LEKTI 15th showed approximately 75 °C.

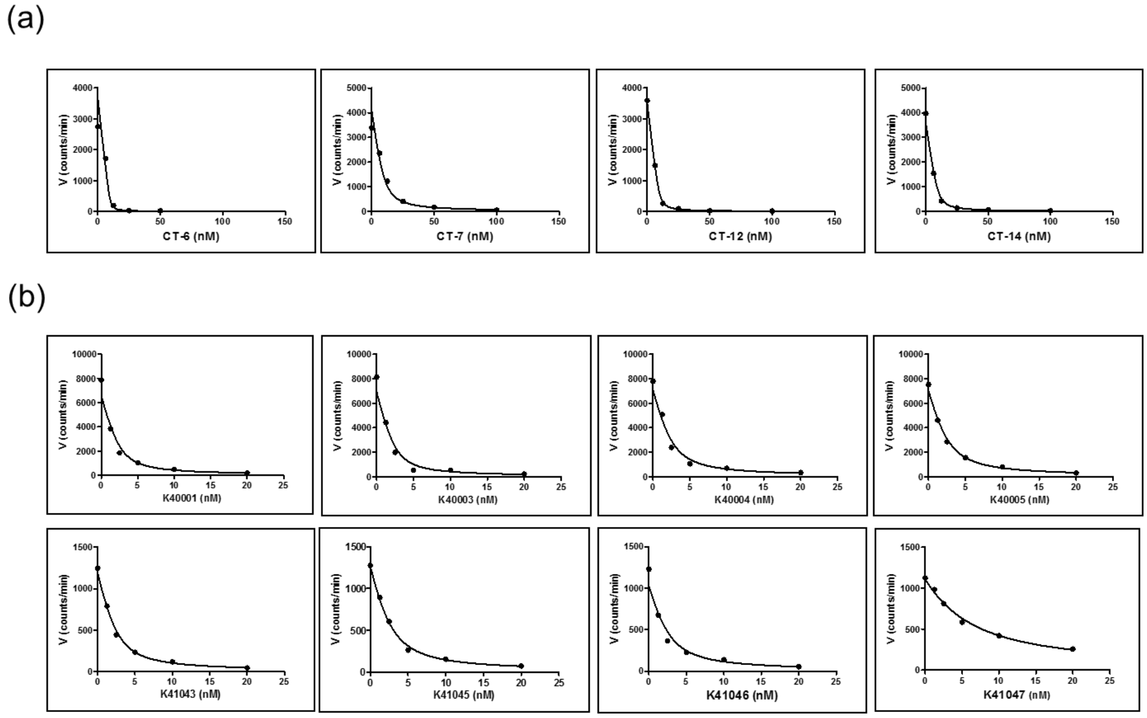

Supplementary Figure 3: Curves used to calculate the inhibitory activity (K_i values) of inhibitors against (a) chymotrypsin and (b) KLK4. K_i values were determined from the results of three independent experiments by fitting the Morrison equation for tight binding inhibitors to the relative reaction velocity using nonlinear regression in GraphPad Prism 5.0.

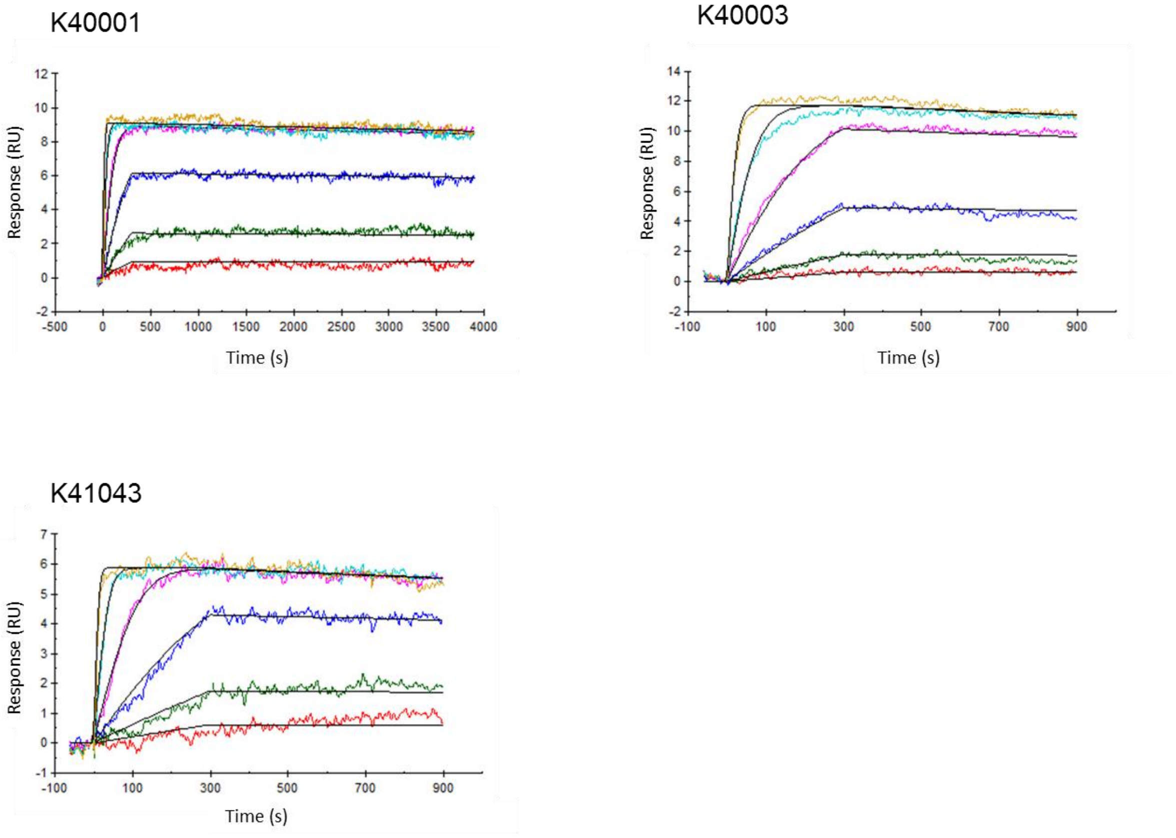

Supplementary Figure 4: Surface plasmon resonance analysis of KLK4 inhibitors binding to immobilized KLK4. Sensorgrams and fitted curves of KLK4 inhibitors are presented. After injecting KLK4 inhibitors (K40001, K40003, and K41043) at 0.08, 0.25, 0.74, 2.22, 6.67, and 20 nM, sensorgrams were analyzed using fitted curves. K_D values were calculated from the determined K_{on} and K_{off} values.

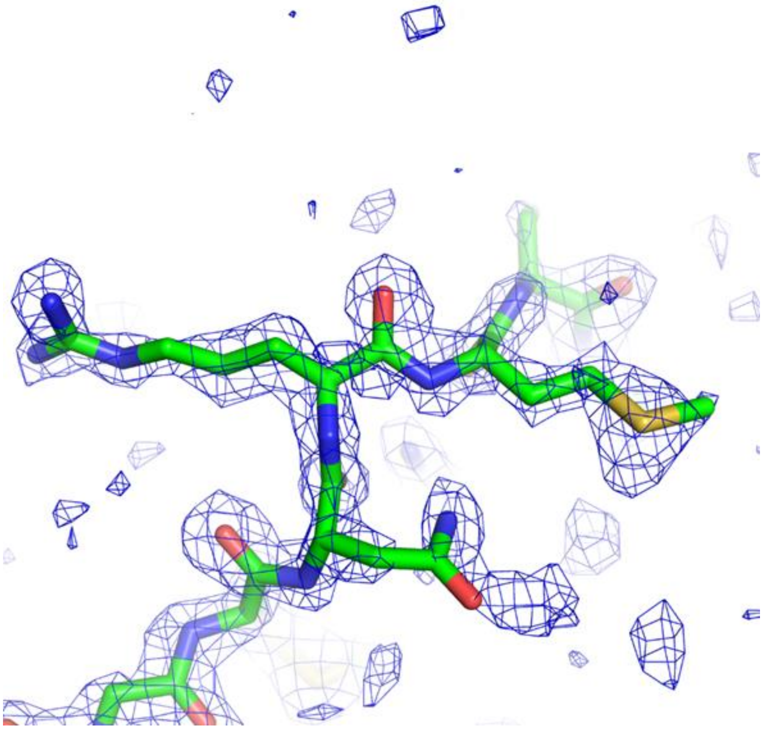

Supplementary Figure 5: Fo-Fc omit map of K41043 (mesh map colored *blue*, contoured at 2.0 sigma) and structural model of K41043 (stick model colored *green*). The map is not interrupted, indicating that K41043 is not a substrate under the crystallization conditions.

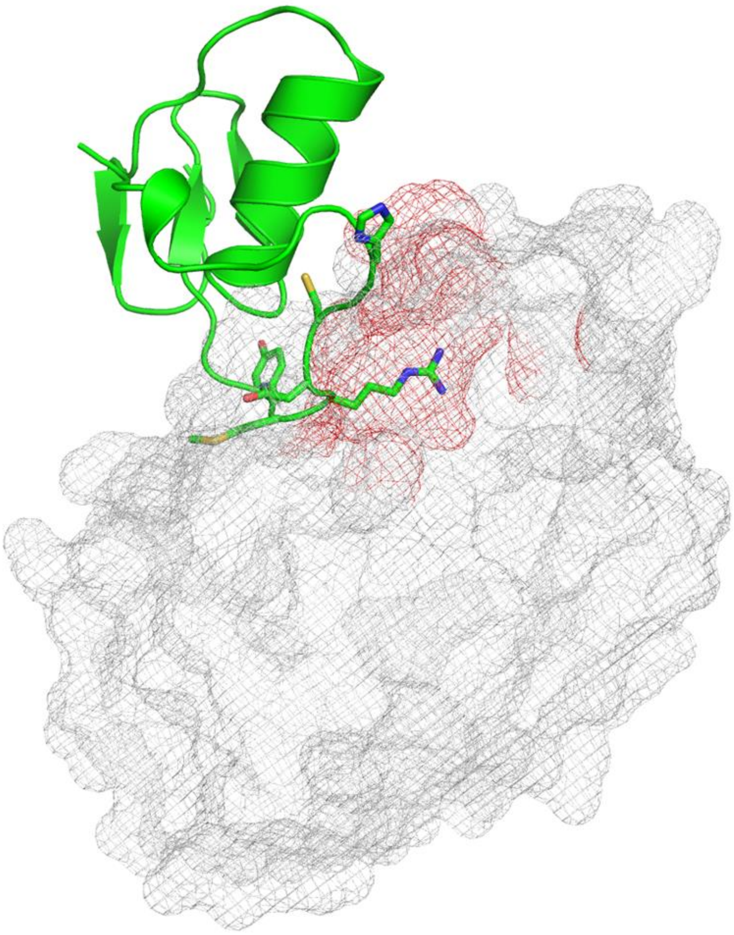

Supplementary Figure 6: X ray crystal structure of the KLK4–K41043 complex. KLK4 which is colored in *gray*, is shown as a mesh model, while K41043 (colored in *green*) is shown as a cartoon model. The *red* region in KLK4 indicates the S1 pocket which is buried by Arg24 of K41043.
